# Combination of farnesyl-transferase inhibition with KRAS G12D targeting breaks down therapeutic resistance in pancreatic cancer

**DOI:** 10.3389/pore.2024.1611948

**Published:** 2024-12-02

**Authors:** Eszter Molnár, Marcell Baranyi, Krisztina Szigeti, Luca Hegedűs, Fanni Bordás, Zsófia Gábriel, Gréta Petényi, József Tóvári, Balázs Hegedűs, József Tímár

**Affiliations:** ^1^ Department of Pathology, Forensic and Insurance Medicine, Semmelweis University, Budapest, Hungary; ^2^ KINETO Lab Ltd., Budapest, Hungary; ^3^ Department of Thoracic Surgery, University Medicine Essen - Ruhrlandklinik, University Duisburg-Essen, Essen, Germany; ^4^ Department of Experimental Pharmacology and the National Tumor Biology Laboratory, National Institute of Oncology, Budapest, Hungary

**Keywords:** PDAC, G12D mutant KRAS, KRAS inhibitor resistance, FTI, combination therapy

## Abstract

Pancreatic adenocarcinoma is one of the deadliest forms of cancer with no effective therapeutic options. A KRAS mutation can be found in up to 90% of all pancreatic tumors, making it a promising therapeutic target. The introduction of new KRAS inhibitors has been a milestone in the history of KRAS mutant tumors; however, therapeutic resistance limits their efficacy. Thus, new therapeutic options, including combination therapies, are urgently needed. Recently, we have shown that KRAS G12C inhibitors in combination with farnesyl-transferase inhibitors exert synergistic antitumor effects. Here, we provide evidence for the feasibility of this combinational approach to break down resistance in KRAS G12D mutant pancreatic cancer. Although we have shown that the 3D environment dramatically sensitizes cells to MRTX1133 treatment, the synergistic effect of this drug combination is present in both 2D and 3D in the PANC1 pancreatic adenocarcinoma model, which showed high resistance to MRTX1133 in 2D. The effects of the combination treatment show an association with the inhibition of farnesylated regulatory proteins, including HRAS and RHEB, along with the expression level of KRAS. Our study warrants further investigation for the potential applicability of KRAS G12D inhibitors in combination with farnesyl-transferase inhibitors for the treatment of KRAS mutant pancreatic adenocarcinoma.

## Introduction

Pancreatic adenocarcinoma (PDAC) is one of the deadliest cancer types, with a 5-year survival rate of only 10% [1]. Due to the lack of specific early symptoms, the majority of the patients are diagnosed at an advanced disease stage with already established metastasis. Moreover, mostly due to the lack of targetable molecular alterations, surgical removal and conventional chemotherapy are the only available treatment options for PDAC patients [1].

Although a number of driver mutations have been identified in PDAC tumors in addition to several tumor suppressors [CDKN2A (cyclin-dependent kinase inhibitor 2A), TP53 (tumor protein p53)] and the predominant KRAS (Kirsten Rat Sarcoma) mutations, the percentage of patients with therapeutically targetable mutations is low [2]. One exception is the relatively rare BRCA1/2 (Breast Cancer type 1/2 susceptibility protein) mutation which renders tumor cells sensitive to PARP [Poly (ADP-ribose) polymerase] inhibitors [3]. Furthermore, PDACs are generally stroma-rich, “cold” tumors with low immunogenicity, in which immunotherapeutic modalities provide no clinical benefit [4] with the exception of the rare occurrence of MMR deficiency which results in sensitivity to immunotherapy [5].

Approximately 90% of PDAC tumors harbor an activating mutation in the KRAS proto-oncogene [6], among which the most frequently present is the G12D mutation at ∼50%, G12V at ∼30% and G12R at 10% while other mutations do not reach the frequency of 5% [7]. In PDAC, G12D mutant KRAS is considered to be a major driver in contrast to lung or colorectal cancer [8]. Mutant KRAS has long been considered “undruggable” due to its compact structure and specific molecular properties, but the recent introduction of allele-specific inhibitors has challenged this label [9]. Covalent inhibitors of the G12C mutant KRAS have already been approved for clinical application [10]. Nevertheless, the vast majority of G12C mutations are mainly linked to lung adenocarcinoma [7]. Clinically available therapeutic options for tumors with other forms of KRAS mutations are yet to be developed. Notably, there are several established KRAS G12D inhibitors available, and even the first reports on the possibility of covalent targeting of KRAS G12D have been published [11]. Among the new KRAS G12D inhibitors, MRTX1133 is the first-in-class specific, non-covalent inhibitor of the KRAS G12D oncoprotein [12]. This small-molecule inhibitor shows high selectivity for the G12D form of KRAS over the wild-type protein, preventing effector protein binding and blocking subsequent cell signaling activation [13]. MRTX1133 can inhibit the viability of KRAS G12D mutation harboring cell lines at nanomolar concentrations and it also shows potent *in vivo* activity [13].

The treatment of KRAS-mutant tumors has been dramatically shifted with recent advances in targeted therapies. However, evidence of acquired or *de novo* resistance to covalent G12C inhibitors has already been demonstrated, with only approximately 40% of patients showing partial response to KRAS-targeting monotherapies in lung adenocarcinoma and less than 20% in colorectal cancer [14]. Several resistance mechanisms have been already described, ranging from new mutations in the KRAS gene to the rewiring of the tumor cells’ signalization network [14, 15]. The latter is most evident in the feedback reactivation of RAS signaling by wild-type RAS proteins (HRAS, NRAS, or the wild-type KRAS allele in the case of heterozygous KRAS mutations) [15]. Additionally, resistance mechanisms revealed in KRAS G12C mutant tumors have been observed following MRTX1133 treatment in G12D mutant cancer models. Upstream activation of ERBB (erythroblastic leukemia viral oncogene homolog) receptors after MRTX1133 exposure has also been shown in colorectal and pancreatic cancers, leading to sustained wild-type RAS signaling [16–18]. Upregulation of mammalian target of rapamycin (mTOR) signaling, epithelial-to-mesenchymal transitions, or specific gene amplifications have also been observed [19].

Thus, several combination approaches have been proposed, most of which focus on the concomitant targeting of mutant KRAS and reactivation of wild-type RAS signaling. These include the combination of KRAS G12C inhibitors with therapeutic modalities targeting upstream elements of the RAS pathway like EGFR (epidermal growth factor receptor) -blocking antibodies or SOS1 (son of sevenless 1) inhibitors in addition to targeting SHP2 (Src homology 2-containing protein tyrosine phosphatase 2), which is also a positive modulator of RAS signaling [20]. The application of immunomodulatory therapies, cell cycle inhibitors, or horizontal targeting of the PI3K-AKT pathway in combination with KRAS G12C inhibitors is also being investigated [20]. As the more effective, covalent targeting of G12D is much harder than in the case of the G12C mutant KRAS, and the first-in-class drug MRTX1133 is also a non-covalent drug, the application of combinational therapy seems to be rational.

Our group has previously shown that the combination of KRAS G12C inhibitors with farnesyl-transferase inhibitors exerts synergistic anti-tumoral effects in several KRAS G12C tumor models, including pancreatic tumor cells [21]. As farnesylation is a key post-translational modification of multiple important regulatory proteins, the addition of farnesyl-transferase inhibitors appears to integrate the advantages of agents used in various KRAS combination therapies. These include inhibition of feedback reactivation of HRAS, horizontal targeting of the PI3K-AKT pathway by RHEB (ras homolog enriched in brain), and cell cycle arrest by inducing defects in the nuclear lamina and CENPE/F (centromere associated protein E/F) proteins [21].

Here, we describe that this combination strategy facilitates the anti-tumoral activity of KRAS G12D inhibition and successfully breaks down therapeutic resistance in pancreatic adenocarcinoma.

## Materials and methods

### Cell culture conditions

PANC1, ASPC1, and SW1990 human pancreatic adenocarcinoma cell lines were obtained from ATCC (The American Type Culture Collection). Cells were cultured in normal tissue culture flasks in DMEM (Dulbecco’s Modified Eagle’s Medium) (Capricorn Scientific, Germany, Ebsdorfergrund) supplemented with 10% FBS (fetal bovine serum) (Euroclone, Italy, Pero) and 1% Penicillin-Streptomycin cocktail (Biosera, France, Cholet) in atmospheric O_2_ and 5% CO_2_ level at 37°C in a cell culture incubator in a humidified atmosphere.

### SRB assay

On the first day of the experiment, cells at 80%–90% confluence were trypsinized (Capricorn Scientific, Germany, Ebsdorfergrund) and counted using a Luna II Automated Cell Counter (Logos Biosystems, South Korea, Anyang). Cells were seeded at a density of 2,500–5,000 cells/well in 300 µL on a 48-well plate depending on the growth rate of each cell line. The next day, an additional 100 µL of medium containing four times the final concentration of MRTX1133 (Medchemexpress, Sweden, Sollentuna) was added to the wells. After a 6-day treatment, the wells were washed with DPBS (Dulbecco’s Phosphate Buffered Saline Solution) (Capricorn Scientific, Germany, Ebsdorfergrund) and the cells were fixed with 10% trichloroacetic acid (TCA) (Reanal, Hungary, Budapest) for 1 h at 4°C. After fixation, TCA was removed, and the wells were serially washed with tap water followed by 15 min of incubation with 0.04% SRB (Sulforhodamine B) dye (Merck, Germany, Darmstadt) dissolved in 1% acetic acid. After staining the cells with SRB, the excess dye was removed, and the cells were washed with 1% acetic acid. Cell-bound SRB was then dissolved in 10 mM Tris buffer and was measured using a Biotek EL800 spectrophotometer (Agilent, United States, Santa Clara) at a wavelength of 570 nm. Measurement data were averaged and normalized using control values and graphed using GraphPad Prism 5 software (GraphPad Software, United States, Boston). Each treatment group contained 3 technical replicates, and the experiments were carried out in 3 independent biological replicates.

### 2D combination tests

For 2D combination tests, PANC1 cells were trypsinized, counted, and seeded onto 48-well plates at a density of 2,500 cells/well. The next day, following the attachment of cells, the wells were treated with fresh medium containing MRTX1133 (Medchemexpress, Sweden, Sollentuna) and tipifarnib (Medchemexpress, Sweden, Sollentuna) at various concentrations and their combinations for 6 days. At the end of the treatment, cells were fixed and quantified as described in the SRB assay section. The experiments were carried out in 3 independent biological replicates.

### 3D combination tests

3D combination tests were performed as previously described [21]. Briefly, PANC1 cells were seeded at 1,000 cells/well on the inner 60 wells of a polyHEMA-coated [Poly (2-hydroxyethyl methacrylate)] (Merck, Germany, Darmstadt) round bottom 96-plate, and left for spheroid formation for a week. Following the formation of regular, compact spheroids 150 µL of fresh medium was added to the cells containing MRTX1133 and tipifarnib alone or in combination at various concentrations for 6 days. On the last day of the experiment, 10 µL of CCK8 reagent (Cell counting kit 8) reagents (Merck, Germany, Darmstadt) were added to each well containing the spheroids and to blank wells. After 24 h of incubation, the optical density was measured using a Biotek EL800 spectrophotometer (Agilent, United States, Santa Clara) at a wavelength of 450 nm. Values for blank wells were extracted from each data set, which were later normalized to control wells and plotted using GraphPad Prism 5 software (GraphPad Software, United States, Boston). Each treatment group contained 3 technical replicates, and the experiments were carried out in 3 independent biological replicates.

### Western blot analyses

Activation of proteins in the PI3K/Akt pathway or the RAF/MEK/ERK cascade along with expression of regulatory proteins and apoptosis/proliferation markers were investigated by Western blot analysis. Briefly, cells were seeded at a density of 300,000 cells/well in 6-well plates and were treated on the next day with 100 nM MRTX1133 (Medchemexpress, Sweden, Sollentuna) and/or 500 nM tipifarnib (Medchemexpress, Sweden, Sollentuna) for 48 h. Following treatment, wells were washed with DPBS (Capricorn Scientific, Germany, Ebsdorfergrund), and cells were fixed with 6% TCA (Reanal, Hungary, Budapest) for 1 h at 4°C. Following fixation, cells were mechanically harvested and centrifuged at 10,000 RCF (relative centrifugal force) for 10 min. The supernatant was then discarded and the centrifuged protein was dissolved in a modified Läemmli-type sample buffer containing 0.02% bromophenol blue (Merck, Germany, Darmstadt), 10% glycerol (Merck, Germany, Darmstadt), 2% SDS (sodium-dodecyl-sulfate) (Merck, Germany, Darmstadt), 100 mM dithiothreitol (DTT) (Merck, Germany, Darmstadt), 5 mM EDT (Merck, Germany, Darmstadt) A, 125 mg/mL urea (Merck, Germany, Darmstadt), 90 mM Tris-HCl, pH 7.9. For the determination of protein concentration, a Qubit 4 fluorometer (Thermo Fisher Scientific, United States, Massachusetts, Waltham) was used according to the manufacturer’s recommendations. For electrophoretic separation, equal amounts of protein (25 µg/lane) were loaded onto 10% polyacrylamide gels (Bio-Rad Laboratories, United States, California, Hercules). The separated proteins were transferred to PVDF (polyvinylidene fluoride) membranes (Thermo Fisher Scientific, United States, Waltham).

Analyses of protein expression/activation were performed using the following antibodies: p-AKT (4058) (Cell Signaling Technology, United States, Massachusetts, Danvers), AKT (9272) (Cell Signaling Technology, United States, Massachusetts, Danvers), p-S6 (2215) (Cell Signaling Technology, United States, Massachusetts, Danvers), S6 (2217) (Cell Signaling Technology, United States, Massachusetts, Danvers), p-ERK1/2 (9101) (Cell Signaling Technology, United States, Massachusetts, Danvers), ERK1/2 (9102) (Cell Signaling Technology, United States, Massachusetts, Danvers), RHEB (13879S) (Cell Signaling Technology, United States, Massachusetts, Danvers), PARP (9542) (Cell Signaling Technology, United States, Massachusetts, Danvers),p-Histone H3 (9701) (Cell Signaling Technology, United States, Massachusetts, Danvers), (Cell Signaling Technology, United States, Danvers) KRAS4B (WH0003845M1) (Merck, Germany, Darmstadt), and HRAS (18295-1-ap) (Proteintech, United Kingdom, Manchester). All antibodies were dissolved according to the manufacturer’s instructions in 5% BSA or dried milk in 1x TTBS (tris-buffered saline supplemented with 1% Tween80) buffer. Membranes were blocked in Everyblot buffer (Bio-Rad Laboratories, United States, California, Hercules) for 5 min at room temperature. Following blocking, the membranes were incubated in primary antibodies overnight at 4°C. Horseradish peroxidase (HSP) conjugated rabbit or mouse secondary antibodies (Cell Signaling Technology, United States, Massachusetts, Danvers) (1:5,000, 1 h, room temperature), and WesternBright ECL (Advansta, United States, California, San Jose) were used for visualization with light-sensitive films. For total proteins, membranes were stripped by boiling in distilled water. Ponceau staining was used for normalization. Quantification was performed using ImageLab software (Bio-Rad Laboratories, United States, California, Hercules). Each cell line was analyzed in 3 biological replicates.

### Cell cycle investigation

**FIGURE 1:**
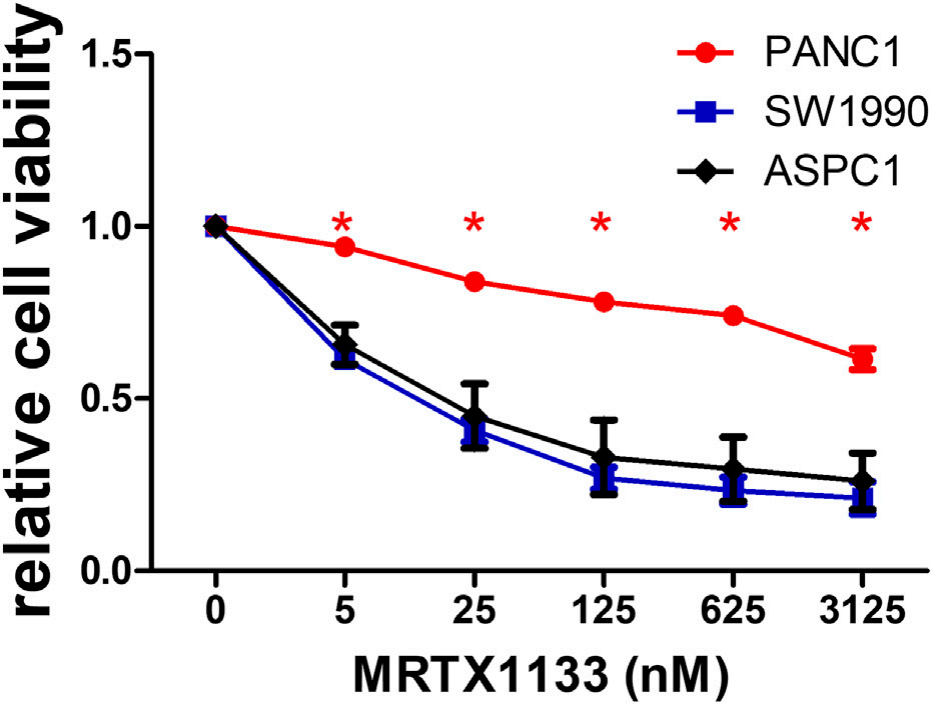
2D cell viability data of pancreatic adenocarcinoma cell lines. Cells were treated with various concentrations of MRTX1133 for 6 days. Control-normalized data show the resistant PANC1 cell line and the sensitive SW1990 and ASPC1 cells. Data were derived from three independent experiments and graphed using GraphPad Prism 5 (n = 3, average ± SEM). Asterisks mark significant differences (*p* < 0.05) measured by two-way ANOVA with Bonferroni post-test, comparing viability data of SW1990 and ASPC1 to PANC1.

Determination of DNA content in each cell was used to evaluate the number of cells in each cell cycle phase as previously described [21]. Briefly, 500 nM tipifarnib (Medchemexpress, Sweden, Sollentuna), 100 nM MRTX1133 (Medchemexpress, Sweden, Sollentuna), or their combination were used for a 96-hour-long treatment. Cells were trypsinized, fixed with ice-cold 70% ethanol and lysed, followed by DAPI (4′,6-diamidino-2-phenylindole) (Chemometec, Denmark, Allerod) staining for 5 min at 37°C. A stabilization buffer was then added, and 10 µL of each sample was loaded onto an 8-well NC slide (Chemometec, Denmark, Allerod). NucleoCounter NC-3000™ system (Chemometec, Denmark, Allerod) was used for quantification of cellular fluorescence (Figure 1).

## Results

### Sensitivity of pancreatic adenocarcinoma cells to MRTX1133 treatment

Three KRAS G12D mutant human pancreatic adenocarcinoma cell lines were tested in conventional 2D cultures for sensitivity to MRTX1133, a specific non-covalent inhibitor of the KRAS G12D protein. While two of them (SW1990 and ASPC1, both harboring a homozygous KRAS G12D mutation) showed high sensitivity to MRTX1133 (IC50 values are 11 and 21 nM, respectively), heterozygous PANC1 was found to be highly resistant (IC50 > 3,125 nM).

### Changes in KRAS-related signaling after MRTX1133 treatment

**FIGURE 2:**
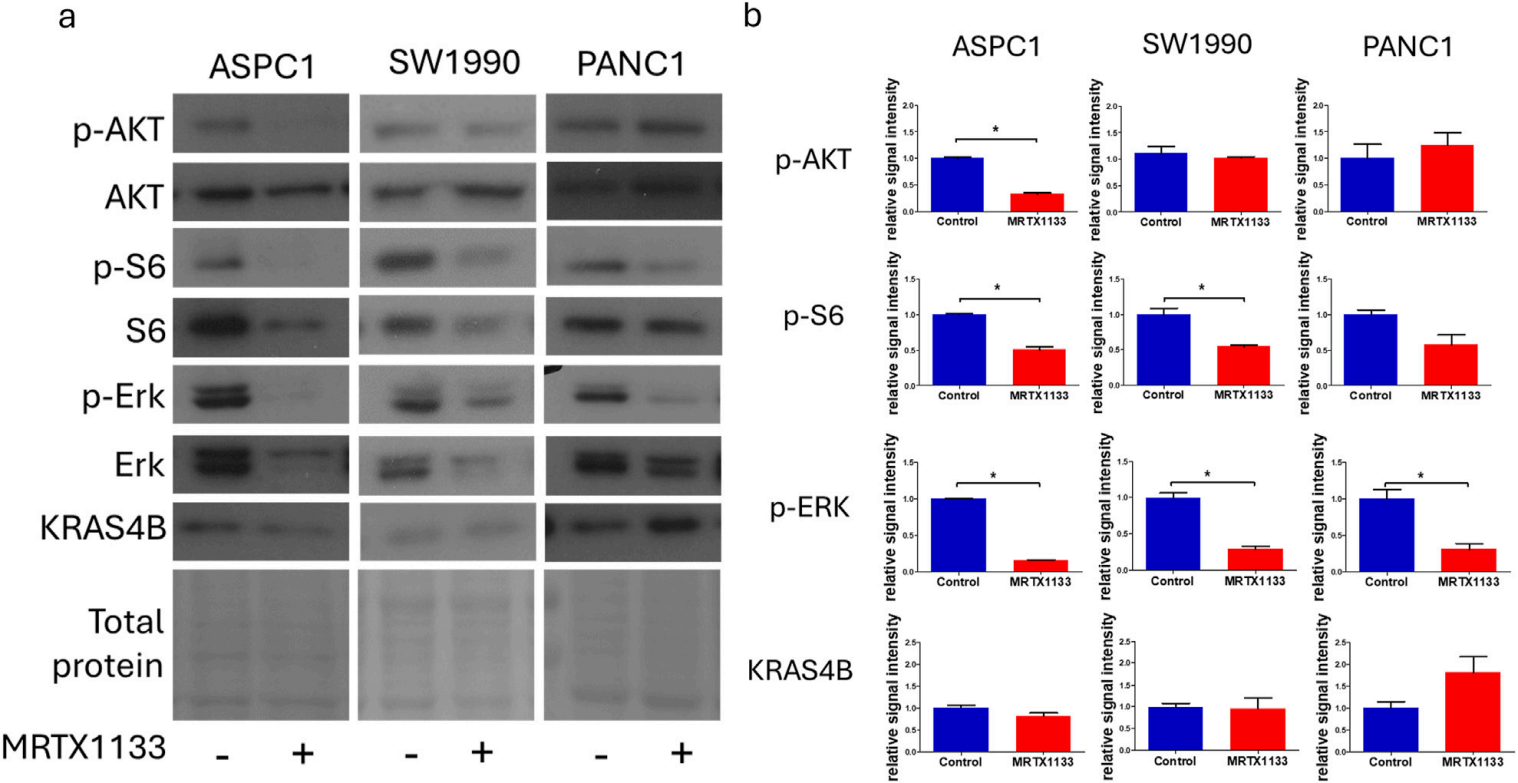
Changes in cell signaling after MRTX1133 treatment. Cells were treated with 100 nM MRTX1133 for 48 h. RAS-related cell signaling was determined by Western blotting. **(A)** Representative bands of the proteins investigated. Activated, phosphorylated proteins are marked with the “p-” prefix. **(B)** Densitometric evaluation of the activation or expression of the represented proteins. Data were normalized to total protein level and expressed relative to control using GraphPad Prism 5 software (±SEM). Data are derived from three independent experiments. Asterisks mark significant differences (*p* < 0.05) as determined by an unpaired *t*-test.

Next, we examined changes in KRAS-related signaling following 48 h of treatment with 100 nM MRTX1133. Strong inhibition of ERK activation could be observed in all cell lines, independently of cell sensitivity data, and a more modest, but still pronounced reduction in S6 phosphorylation in all cell lines tested, although the reduction in the PANC1 cell was not statistically significant (*p* = 0.11) (Figure 2). Interestingly, activation of AKT, an upstream member of the PI3K-AKT-mTOR signaling pathway of which S6 is a downstream element, was significantly reduced in the ASPC-1 cell line after MRTX1133 treatment. Importantly, the expression level of KRAS4B was elevated in the MRTX1133-resistant PANC1 cell line following treatment, although this increase was not statistically significant (*p* = 0.17).

### A combination of farnesyl-transferase inhibitors with MRTX1133 shows synergistic anti-tumoral effects in the resistant cell line

**FIGURE 3:**
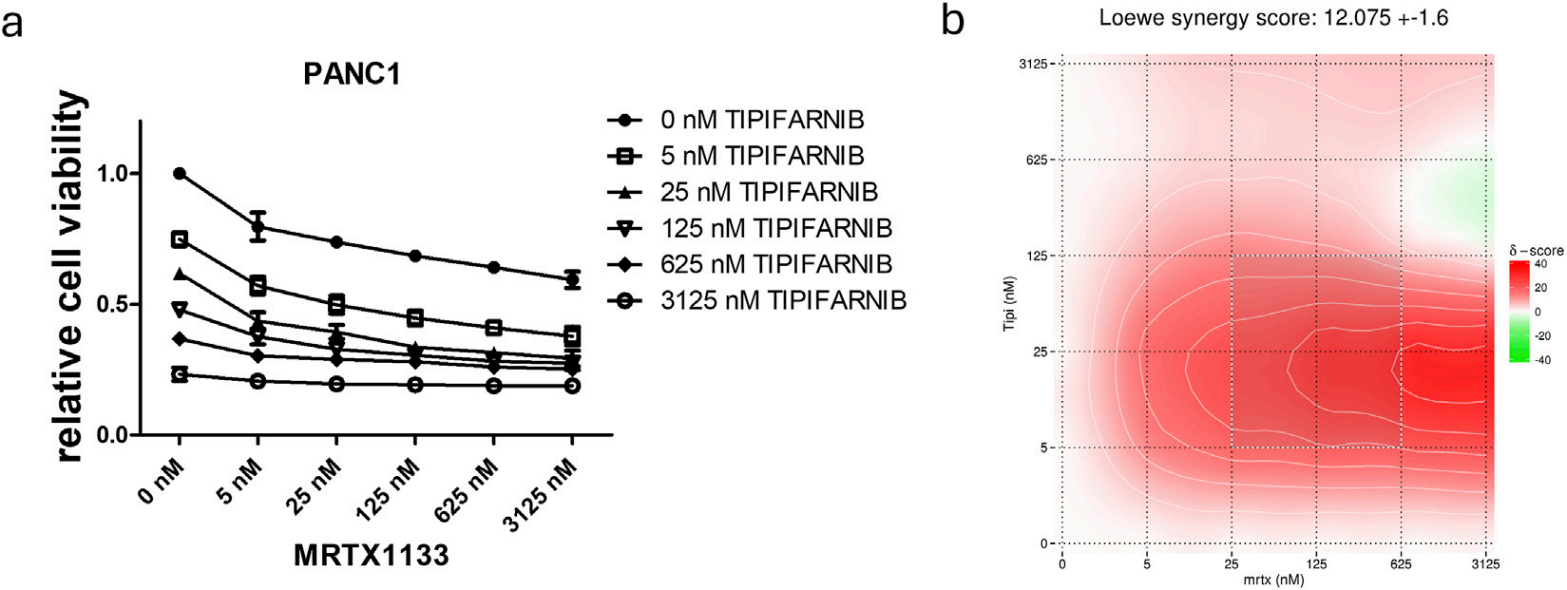
Combination of farnesyl-transferase inhibition with KRAS G12D targeting in MRTX1133 resistant PANC1. 6-day combination therapy of tipifarnib and MRTX1133 was applied to 2D cultures of PANC1 cells. **(A)** Control-normalized viability values. Data are derived from three independent experiments and are expressed relative to control (n = 3, ±SEM) **(B)** Synergy map calculated from viability results using synergyfinder.org. In general, a synergy score greater than 10 is considered to have synergistic effects. Note that the most synergistic area is at low dose tipifarnib and high dose MRTX1133.

We also focused on the PANC1 cell line, which showed high resistance to KRAS targeting. Previously, we had demonstrated the synergistic effects of farnesyl-transferase inhibitors with KRAS G12C inhibitors, so we investigated whether they could potentiate the effect of MRTX1133 to break down resistance. Notably, the combination of tipifarnib with MRTX1133 resulted in strong synergistic anti-tumor effects with a Loewe synergy score of 12 based on the website^1^ (Figure 3) [22]. Synergy scores greater than 10 are considered to have synergistic effects. Notably, the synergy map revealed that the highest synergy occurred when low-dose tipifarnib and high-dose MRTX1133 were applied. Additionally, combining an alternative farnesyl-transferase inhibitor, lonafarnib, with MRTX1133 also resulted in an average Loewe synergy score of 6.182. Of note, the combination of MRTX1133 with high-dose lonafarnib showed strong synergistic interactions as visualized in the synergy map in Supplementary Figure S1.

### PANC1 cells showed enhanced sensitivity to single agent MRTX1133 in 3D spheroid model

**FIGURE 4:**
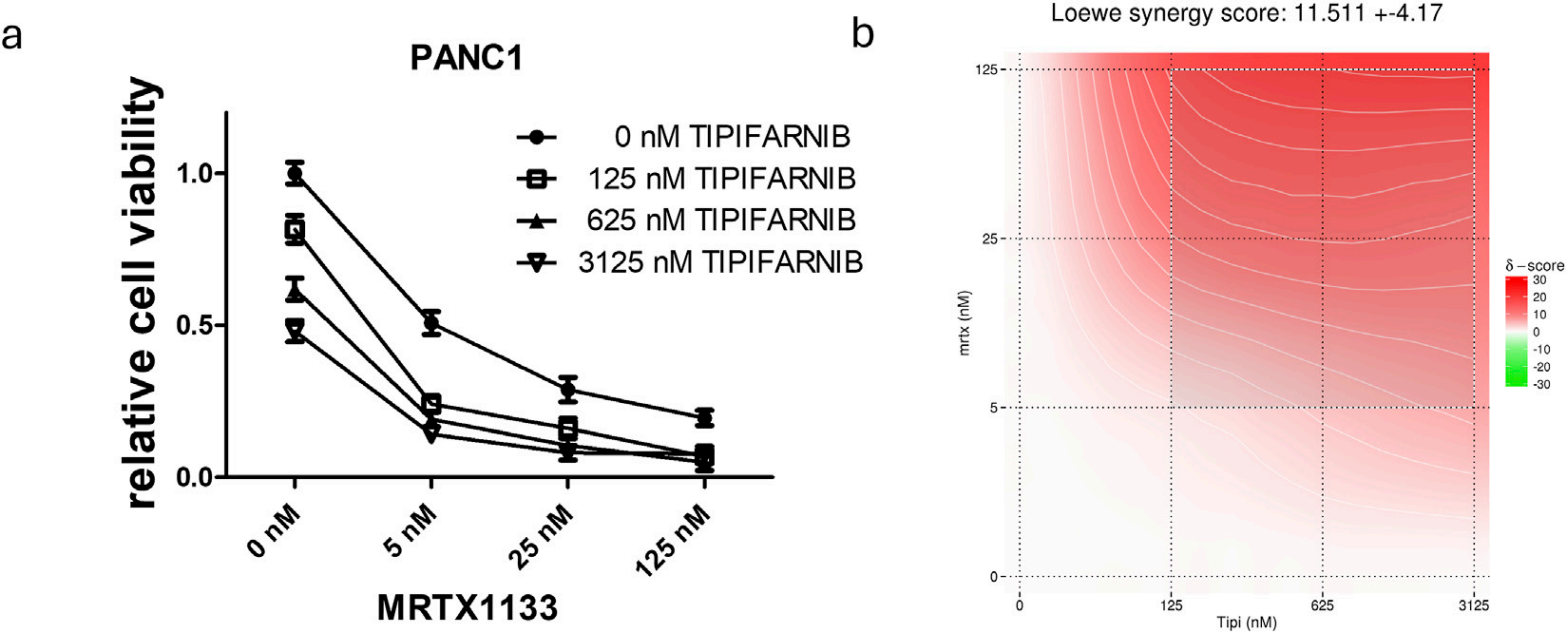
Combination of farnesyl-transferase inhibition with KRAS G12D targeting in PANC1 spheroids. A 6-day-long combination therapy of tipifarnib and MRTX1133 was applied to spheroid cultures of PANC1 cells. **(A)** Control-normalized viability values. Data are derived from three independent experiments and are expressed relative to control (n = 9, ±SEM) **(B)** Synergy map calculated from viability results using synergyfinder.org. Generally, a synergy score greater than 10 is considered to have synergistic effects. Note that the most synergistic area is at high dose tipifarnib and high dose MRTX1133.

Next, we intended to examine the effects of single and combination treatment in a more complex, spheroid-based model in a 3D environment. Notably, we observed that sensitivity to MRTX1133 already as a single agent was greatly enhanced (Figure 4). Changes in MRTX1133 sensitivity could not be tested in the SW1990 and ASPC1 cell lines, as these cells did not show growth in 3D conditions in our settings (data not shown). The combination of MRTX1133 with tipifarnib resulted in synergistic drug interactions in 3D conditions with a Loewe synergy score of 11.5.

### Changes in RAS-related cell signaling in PANC1 cells after combination therapy

**FIGURE 5:**
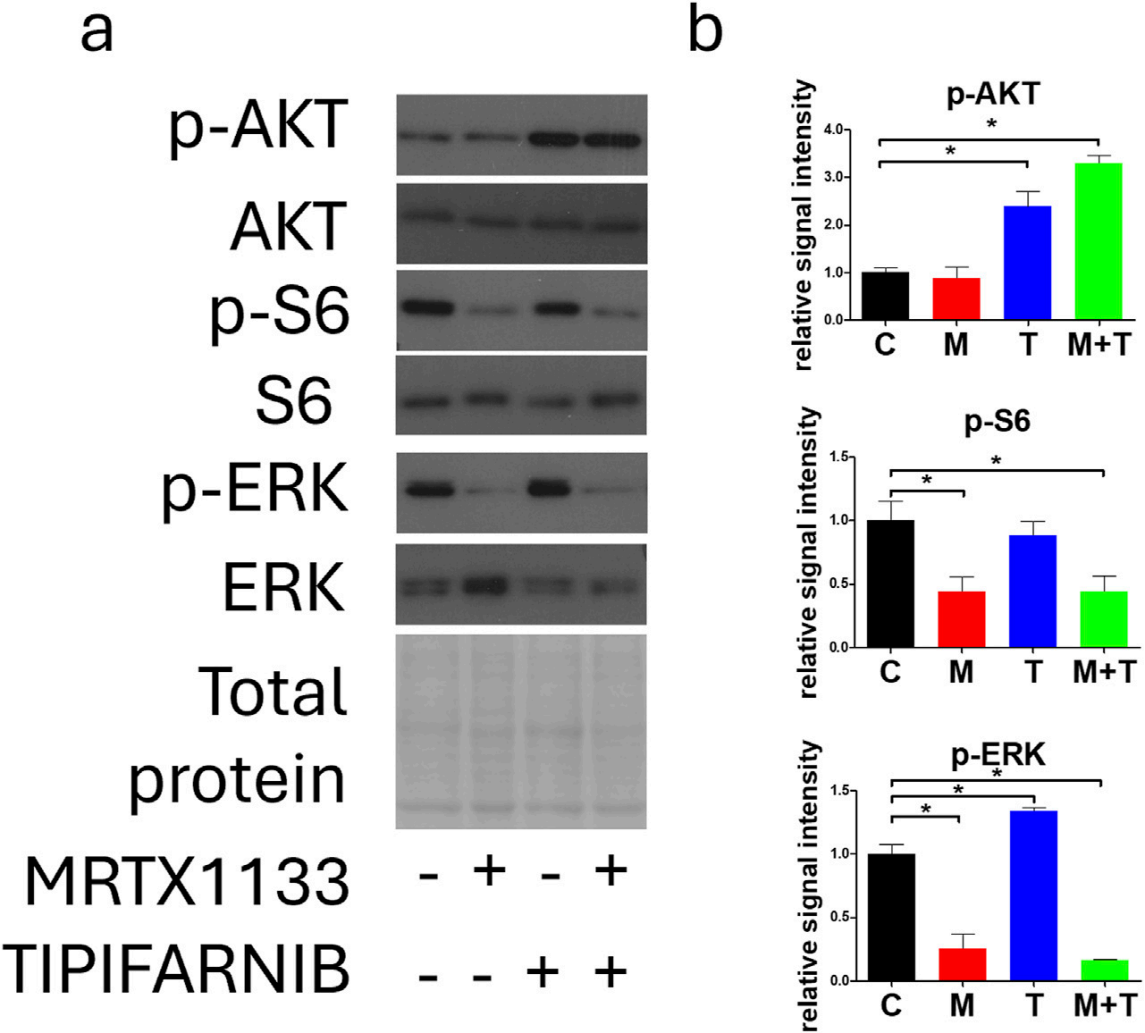
Changes in cell signaling after MRTX1133 (M), tipifarnib (T), or combination treatment (M+T). PANC1 cells were treated with 100 nM MRTX1133 and 500 nM tipifarnib alone or in combination for 48 h. RAS-related cell signaling was determined by Western blotting. **(A)** Representative bands of the proteins investigated. Activated, phosphorylated proteins are marked with the “p-”prefix. **(B)** Densitometric evaluation of the activation of the represented proteins. Data were normalized to total protein and expressed relative to the control and graphed with GraphPad Prism 5 software (“C”, ±SEM). Data are derived from three independent experiments. Asterisks mark significant differences (*p* < 0.05) tested with one-way ANOVA followed by Bonferroni’s Multiple Comparison test comparing treatment groups to control samples.

We also investigated the effects of combination therapy on RAS signaling (Figure 5). As described above, MRTX1133 single-agent treatment strongly reduced the activation of S6 and ERK but evoked no change in the phosphorylation of AKT. Interestingly, both tipifarnib alone and in combination with MRTX1133 strongly increased AKT phosphorylation in PANC1 cells. Tipifarnib alone significantly increased ERK phosphorylation but it did not alter S6 activation. Combination treatment substantially reduced both S6 and ERK activation.

### Effects of combination therapy on apoptosis, proliferation, and farnesylation in PANC1 cells

**FIGURE 6:**
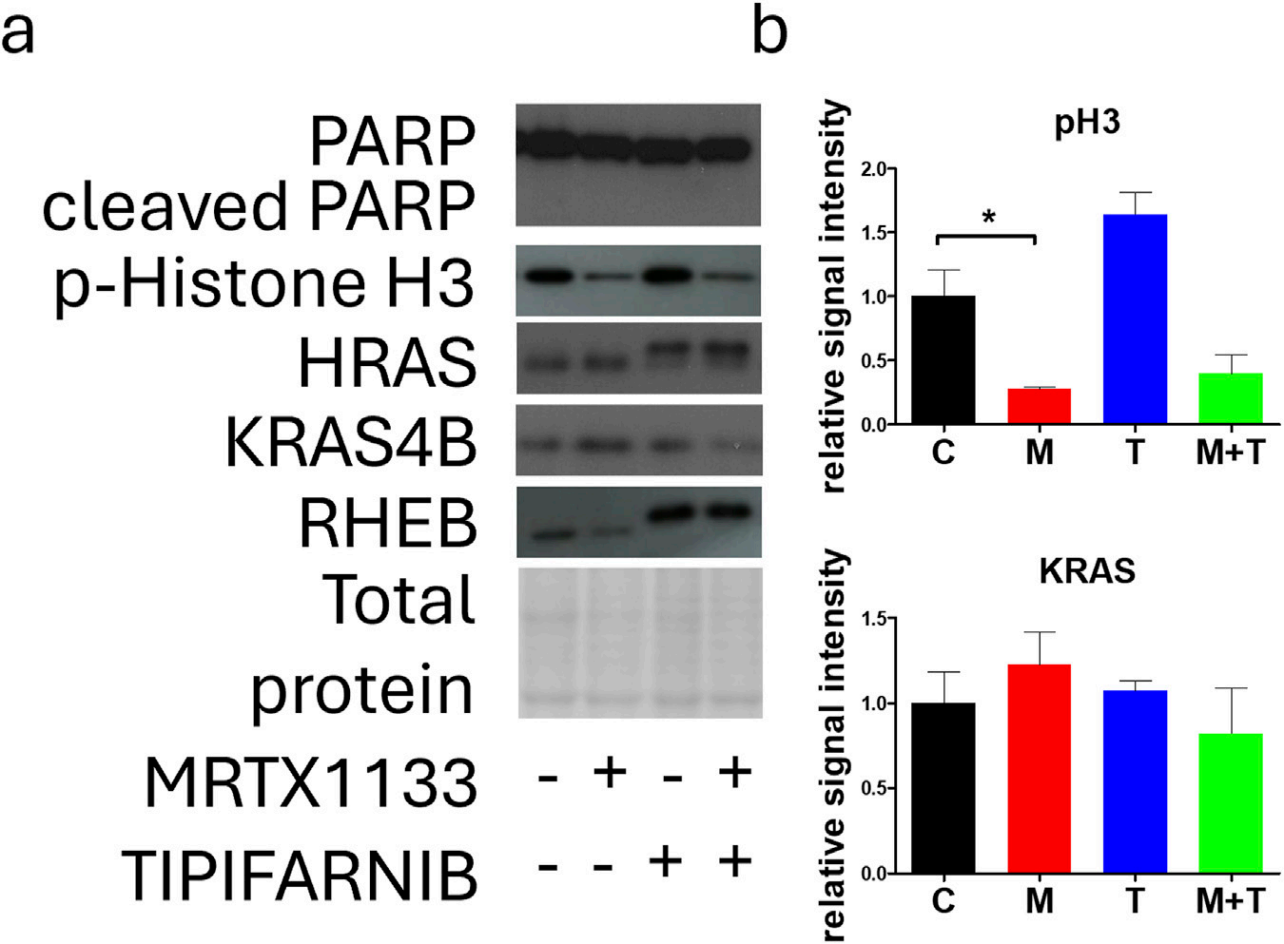
Effects of MRTX1133 (M), tipifarnib (T), or combination(M+T) treatment on apoptosis, proliferation, and farnesylation. PANC1 cells were treated with 100 nM MRTX1133, 500 nM tipifarnib, or their combination for 48 h. RAS-related cell signaling was determined by Western blotting. **(A)** Representative bands of the proteins investigated. **(B)** Densitometric evaluation of the activation or expression of the represented proteins. Data were normalized to total protein and expressed relative to the control (C) and graphed with GraphPad Prism 5 software (n = 3, ±SEM). Data are derived from three independent experiments. Asterisks mark significant differences (*p* < 0.05) tested with one-way ANOVA followed by Bonferroni’s Multiple Comparison test comparing treatment groups to control samples.

Next, we examined the effect of MRTX1133, tipifarnib, and combination therapy on apoptosis induction, proliferation, and changes in farnesylation of RAS family members in the PANC1 cell line. Notably, no apoptosis could be detected based on the complete absence of cleaved PARP in all the treatments (Figure 6). We found that the expression of p-Histone H3, a known marker of M-phase cells, was drastically reduced with MRTX1133 treatment. Combination treatment also reduced p-Histone H3 expression, although this change was not statistically significant. Interestingly, tipifarnib treatment caused a visible (but not statistically significant) increase in the levels of p-Histone H3. Notably, tipifarnib and the combination induced an upward shift in HRAS and RHEB proteins, which is a well-known sign of farnesylation inhibition. KRAS farnesylation was not affected by any of the treatments as expected. However, clear trends, although not statistically significant could be observed in changes in KRAS expression, where combination therapy reduced the mean level of total KRAS, in contrast to the slight increase seen with MRTX1133 monotherapy.

### Effects of combination therapy on cell cycle distribution of PANC1 cells

**FIGURE 7:**
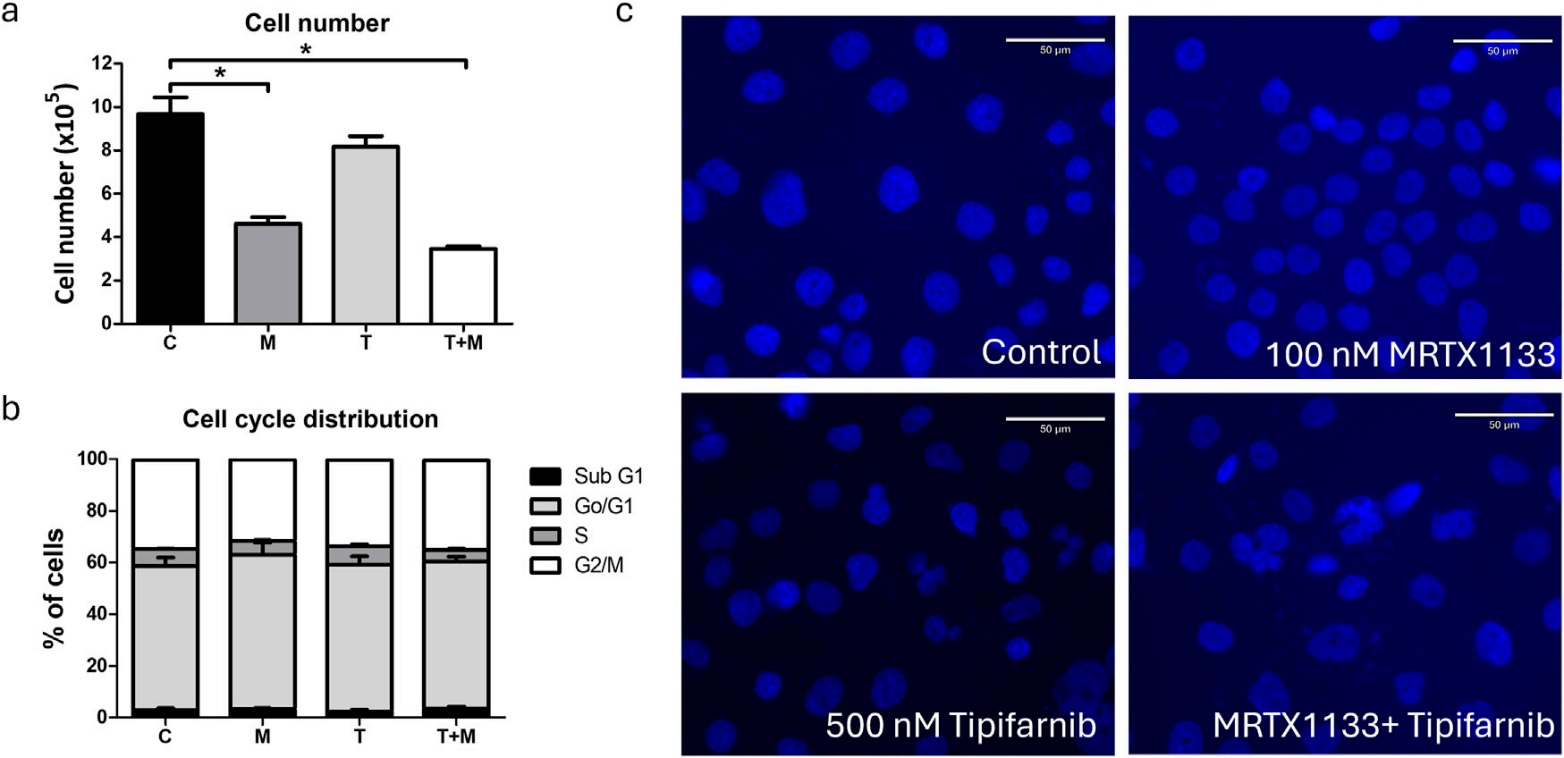
Effects of MRTX1133 (M), tipifarnib (T), or combination (M+T) treatment on cell cycle distribution. Cells were treated with 100 nM MRTX1133, 500 nM tipifarnib, or their combination for 96 h **(A)** Changes in total cell number following treatment. **(B)** Cell cycle distribution of cells following treatment. MRTX1133 and combination therapy reduced the ratio of cells in the S-phase, although these changes are not statistically significant. **(C)** Morphology of cell nucleus following 96 h of treatment. Note the aberrant nuclear morphology after tipifarnib or combination treatment. Scale bar = 50 μm. Data shown in the graphs are derived from three independent experiments and are expressed as mean ± SEM. Asterisks mark significant differences (*p* < 0.05) tested by one-way ANOVA followed by Bonferroni’s Multiple Comparison test comparing treatment groups to control samples.

Next, we examined the effects of combination therapy on cell cycle distribution after a 96-hour treatment. We observed significantly reduced cell numbers after MRTX1133 and combination treatment, consistent with the results of the 2D and 3D combination tests (Figure 7A). However, only numerical, statistically insignificant changes could be observed in the distribution of cells within distinct cell cycle phases. MRTX1133 monotreatment and combination treatment slightly, but not statistically significantly reduced the ratio of S-phase cells and increased the ratio of subG1 cells. Tipifarnib treatment induced no change in cell cycle distribution (Figure 7B). Interestingly, when we investigated the morphology of the DAPI-stained nuclei, we observed a mixed population of PANC1 cells in the control group exhibiting mainly smaller nuclei, in addition to a population of larger, presumably tetra-or polyploid cells. MRTX1133 treatment almost completely eliminated the latter fraction and induced a doughnut-like nuclear morphology that was previously linked with mitotic defects [23]. Notably, tipifarnib and combination treatment resulted in aberrant, lobed, and segmented nuclear morphology (Figure 7C).

## Discussion

In the past few years, intensive work by the research community has led to breakthroughs in the treatment of KRAS-mutant cancers, especially in the G12C mutant form [20]. KRAS was considered undruggable due to its compact form, picomolar affinity for GTP (Guanosine-5′-triphosphate), and high homology between the different RAS genes [20]. Therefore, an activating mutation in this proto-oncogene simply served as an exclusion criterion for any targeted therapy, like EGFR-targeting agents [7]. Thus, the development of allele-specific inhibitors of KRAS represents a major step forward, especially considering the positive responses to these agents observed in the clinic. Although these clinically observed anti-tumoral activities are unprecedented in treating KRAS mutant tumors, the majority of patients still show intrinsic resistance to KRAS inhibitors [14]. For this reason, combination settings are urgently needed to break down resistance and to increase the number of patients that show response. Accordingly, various combination therapies are being tested, including the combination of KRAS G12C inhibitors with EGFR, SOS1, SHP2, and cell cycle inhibitors or horizontal targeting of the AKT-mTOR pathway, the majority of which are currently being investigated in clinical trials [20].

Recently, we have shown that the combination of farnesyl-transferase inhibition with KRAS G12C inhibitors results in synergistic antitumoral effects, independent of the tissue of origin in 2D, 3D, and *in vivo* models [21]. We have demonstrated that the addition of farnesyl-transferase inhibitors to KRAS targeting interferes with multiple signaling mechanisms, like feedback reactivation of RAS signaling through wild-type HRAS proteins, AKT-mTOR signaling by blockade of farnesylated RHEB, or blocking cell cycle progression by interfering with the laminar network and farnesylation of CENPE/F [21]. Notably, most of these mechanisms overlap with the currently investigated combinational approaches targeting oncogenic and wild-type RAS signaling in parallel (KRAS G12C inhibitors plus EGFR, SOS1, or SHP2 targeting) or with inhibitors of the cell cycle or the AKT-mTOR pathway [15, 20].

Here, we have demonstrated that the application of tipifarnib, one of the most potent farnesyl-transferase inhibitors results in synergistic anticancer effects in combination with KRAS G12D targeting pancreatic adenocarcinoma cells that have been shown to be therapy-resistant under 2D conditions.

In our panel of pancreatic adenocarcinoma cell lines, PANC1, a well-known model of human PDAC, showed resistance to MRTX1133 single agent treatment (IC50 > 3,000 nM) in contrast with ASPC1 (IC50 = 21.9 nM) and SW1990 (IC50 = 11.6 nM). In assessing changes in RAS-related signaling after MRTX1133 treatment, we observed strong inhibition of ERK and S6 activation, key members of the two most-studied RAS-regulated pathways. The most unique - although not statistically significant - change in the therapy-resistant cell line was the elevation in KRAS expression after the MRTX1133 application. As this phenomenon could not be observed in the other cell lines, it may reflect a compensatory resistance mechanism.

Next, we sought to examine whether combining tipifarnib with MRTX1133 could overcome the resistance of the PANC1 cell line *in vitro*. Notably, the combination therapy in the 2D model resulted in synergistic anti-cancer effects with a Loewe score of 12. The strongest effect could be seen when the highest MRTX1133 dose was combined with a lower tipifarnib concentration. Additionally, the combination of MRTX1133 with lonafarnib, an alternative farnesyl-transferase inhibitor, also showed synergistic drug interactions, especially at higher FTi concentrations.

We also evaluated the efficacy of combination therapy in PANC1 tumor spheroids. In line with previous findings by others [24], we observed a dramatic change in sensitivity to KRAS targeting when we compared 2D to 3D conditions, more specifically, from IC50 > 3,000 nM in 2D conditions to IC50 = 4.8 nM in the 3D environment. Differential effects of 3D compared to 2D culture conditions are a well-known phenomenon in preclinical cancer research and 3D culture is considered to be more representative of the *in vivo* situation [25, 26]. Notably, it has been shown that culturing cells in spheroids enhances the effects of chemotherapeutic agents [27], or sensitizes towards KRAS targeting [24, 28]. We also have demonstrated in our previous work that KRAS G12C mutant models are more prone to direct KRAS targeting in 3D or *in vivo* compared to 2D monoculture [21]. Unfortunately, the other 2 cell lines were not able to grow into spheroids in our settings, therefore the sensitivity of PANC1 to MRTX1133 could not be directly compared to them in 3D.

Still, the addition of tipifarnib resulted in synergism with a Loewe score of 11.5, although the most synergistic area in the synergy map visualizing synergy scores has shifted to higher tipifarnib concentrations combined with higher MRTX1133 doses.

Examination of RAS signaling revealed that tipifarnib and combination therapy elevated the level of activated AKT while reducing the phosphorylation of S6, which is a downstream target of AKT. This phenomenon can be partly explained by inhibition of RHEB farnesylation as well as crosstalk between the RAS/MEK/ERK pathway and the PI3K-AKT-mTOR signaling [29]. Indeed, we demonstrated that tipifarnib and combination therapy successfully blocked RHEB farnesylation, leading to the accumulation of its non-farnesylated, non-functional form. ERK activation was also significantly increased after tipifarnib treatment; however, combination therapy reduced ERK phosphorylation similar to MRTX1133 monotherapy.

Investigation of HRAS revealed that tipifarnib and combination therapy depleted functional, farnesylated HRAS by interfering with its farnesylation. Previously, we have shown that KRAS targeting results in compensatory reactivation of wild-type RAS proteins, more specifically, overactivation of HRAS induced by KRAS G12C inhibitors [21]. Thus, successful inhibition of its farnesylation can also abrogate this resistance mechanism in KRAS G12D targeting.

We also assessed changes in KRAS expression, although this protein is not sensitive to farnesylation inhibition due to alternative geranyl-geranylation [30]. In line with this, no upward shift could be observed after farnesyl-transferase inhibitor treatment. Interestingly, although not statistically significant, visible trends in changes in KRAS expression could be observed, with the combination treatment reducing KRAS levels not only compared to MRTX1133 monotherapy, which increased them, but also compared to the control. Although the mechanism behind this phenomenon is not known, it may contribute to the observed synergy between the two drugs.

No apoptosis could be detected in any of the treatments based on the level of cleaved PARP. This could be explained by the fact that PANC1 has homozygous mutations in TP53 (p.Arg273His), a key regulator of apoptosis. Interestingly, the expression of p-Histone H3, a known marker of M-phase cells was decreased by MRTX1133 and combination therapy (the latter change was not statistically significant), whereas tipifarnib monotherapy slightly increased its expression. This finding is in line with our previous observation, where we showed that farnesyl-transferase inhibition leads to defects in cell division, leading to the accumulation of M-phase cells [21]. Interestingly, when we analyzed the cell cycle distribution after a longer, 96-hour treatment, we observed a decrease in the S-phase cell ratio after MRTX1133 and combination therapy, with a more pronounced effect of the latter. In line with our previous findings, we found that tipifarnib and combination therapy led to an accumulation of cells with aberrant, segmented nuclear morphology, indicating defects in the mitotic process [21].

In summary, we have demonstrated that MRTX1133 sensitivity shows high variability in KRAS G12D mutant pancreatic adenocarcinoma models in 2D culture. Further investigation of the resistant cell model revealed that the addition of a farnesyl-transferase inhibitor to MRTX1133 can abrogate this resistance, leading to synergistic antitumoral effects. Interestingly, culturing cells in spheroid culture dramatically sensitized resistant cells to MRTX1133 treatment. Nevertheless the synergistic effects of combination treatment are also maintained in the 3D environment.

Regarding the mechanisms behind the synergistic effects, we identified elevated KRAS expression after MRTX1133 treatment, which was ultimately decreased by combination therapy. Additionally, similar to our previous observations, we found that inhibition of farnesylation of RHEB and HRAS may contribute to synergistic effects.

Our findings warrant further investigation of the applicability of KRAS G12D targeting in combination with farnesyl-transferase inhibitors in KRAS G12D mutant pancreatic adenocarcinoma.

## Data Availability

The raw data supporting the conclusions of this article will be made available by the authors, without undue reservation.

## Footnotes

1
https://synergyfinder.org
