# Supplementary material for: Combination of farnesyl-transferase inhibition with KRAS G12D targeting breaks down therapeutic resistance in pancreatic cancer
Source: Pathol Oncol Res. 2024 Dec 2;30:1611948. doi: 10.3389/pore.2024.1611948 (PMC11646715; doi:10.3389/pore.2024.1611948)
Supplement: Supplementary file 1 [file DataSheet1.docx]

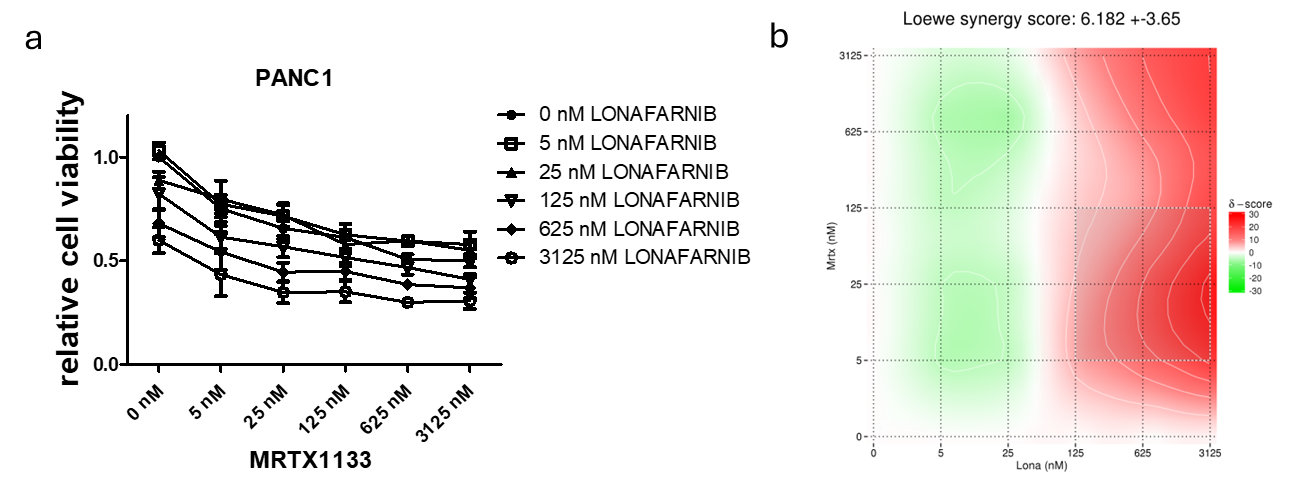


**Supplementary Figure 1. Combination of farnesyl-transferase inhibition with KRAS G12D targeting in MRTX1133 resistant PANC1.** 6-day-long combinational therapy of lonafarnib and MRTX1133 was applied to 2D cultures of PANC1 cells. a) Control-normalized viability values. Data is derived from three independent experiments and is expressed relative to control (n=3, +/- SEM). b) The synergy map is calculated from viability results using synergyfinder.org. Generally, a synergy score higher than 10 is considered to have synergistic effects. Note that the most synergistic area is at high-dose lonafarnib concentrations independent of MRTX1133 concentration.
